# Computational Identification of Four Spliceosomal snRNAs from the Deep-Branching Eukaryote *Giardia intestinalis*

**DOI:** 10.1371/journal.pone.0003106

**Published:** 2008-08-29

**Authors:** Xiaowei Sylvia Chen, W. Timothy J. White, Lesley J. Collins, David Penny

**Affiliations:** Allan Wilson Centre for Molecular Ecology and Evolution, IMBS, Massey University, Palmerston North, New Zealand; Yale University, United States of America

## Abstract

RNAs processing other RNAs is very general in eukaryotes, but is not clear to what extent it is ancestral to eukaryotes. Here we focus on pre-mRNA splicing, one of the most important RNA-processing mechanisms in eukaryotes. In most eukaryotes splicing is predominantly catalysed by the major spliceosome complex, which consists of five uridine-rich small nuclear RNAs (U-snRNAs) and over 200 proteins in humans. Three major spliceosomal introns have been found experimentally in *Giardia*; one *Giardia* U-snRNA (U5) and a number of spliceosomal proteins have also been identified. However, because of the low sequence similarity between the *Giardia* ncRNAs and those of other eukaryotes, the other U-snRNAs of *Giardia* had not been found. Using two computational methods, candidates for *Giardia* U1, U2, U4 and U6 snRNAs were identified in this study and shown by RT-PCR to be expressed. We found that identifying a U2 candidate helped identify U6 and U4 based on interactions between them. Secondary structural modelling of the *Giardia* U-snRNA candidates revealed typical features of eukaryotic U-snRNAs. We demonstrate a successful approach to combine computational and experimental methods to identify expected ncRNAs in a highly divergent protist genome. Our findings reinforce the conclusion that spliceosomal small-nuclear RNAs existed in the last common ancestor of eukaryotes.

## Introduction

Extant eukaryotes are marked by having RNA extensively processing other RNA molecules, whether it is RNase P on tRNAs, RNase MRP and snoRNAs on rRNAs, or snRNAs on mRNAs. In addition RNAi processes are known to inhibit or enhance mRNA expression. A major question in eukaryotic origin is the extent of RNA processing in the last common ancestor of eukaryotes. Perhaps the major question is whether much of the RNA processing traces back to the proposed RNA World [1] and how much is a later invention within eukaryotes [2]. Here we focus particularly on the major spliceosomal snRNAs involved in mRNA splicing, and address the question whether these small snRNAs occur in all deep eukaryotic lineages; in other words, whether the early splicing mechanism in eukaryotes involved both RNA and proteins, or was initially a protein mediated process, with RNAs added later. Here we use a combination of computational techniques with experimental evaluation of the results to help test these alternatives.

The spliceosome is one of the most important RNA-processing units in eukaryotes. The presence of some spliceosomal introns in deep-branching eukaryotes [3]–[5] is consistent with some form of the splicing mechanism having evolved very early during eukaryotic evolution [6]. Eukaryotes can be classified into five main groups [7], although the early branching order of these five groups is yet unknown. *Giardia* belongs to the deep-branching lineage of diplomonads; these are often considered one of the deepest branching lineages of eukaryotes, but little is known in diplomonads of RNA involvement in processing other RNAs. Therefore *Giardia* is particularly important for studying the evolution of major RNA-processing pathways. In general, we followed the approach of Collins and Penny [8] by searching for a feature in deep lineages of eukaryotes, to infer the ancestral state of modern eukaryotes.

To date only three introns have been experimentally confirmed in *Giardia*. The first is a short (35nt) non-canonical intron (5′-CT – AG-3′) located within the mitosomal [2Fe-2S] ferredoxin protein [5], the second a 109nt canonical intron (5′-GT – AG-3′) found in the ribosomal protein Rp17a [4] and the third a 220nt canonical intron found in an unassigned ORF [4]. Some additional introns have been predicted (SW Roy, pers. comm.), but they have not yet been confirmed experimentally. Introns can be both gained and lost during evolution [9] therefore we cannot just assume that the ancestral eukaryotes had very few introns. For example, there appears to be selection for the loss of introns in eukaryotes with a short life cycle [10].

Despite the common assumption that the spliceosome is responsible for the removal of introns in all eukaryotes, the existence and nature of a spliceosome in *Giardia* are at this stage still assumed. A desirable classical approach would be to biochemically extract whole spliceosomes, examine then test the individual components. However, this is an extremely non-trivial exercise even on model eukaryotic spliceosomes, for which a lot is known. Working with non-model organisms is even more difficult. Therefore, a more computational approach is necessary in order to identify good candidates.

Genomic surveys [5], [6] have inferred a number of spliceosomal proteins from the *Giardia* genome. These include homologues of Prp8, Prp11, Prp28 and Prp31; a number of DExH-box RNA-helicases which have homologues in bacteria but which also have important roles in eukaryotic intron splicing; 11 archaeal-like Sm and Lsm core peptides which coat the spliceosomal snRNAs; and a number of U-snRNA-specific peptides. It is therefore very likely that *Giardia* has a functional major spliceosome, but to date there have been no biochemical studies on the entire spliceosome or any of the snRNAs that comprise its catalytic core.

In humans, the major spliceosome is composed of over 200 proteins and five uridine-rich small nuclear RNAs (U1, U2, U4, U5 and U6) that form dynamic protein-RNA and RNA-RNA interactions [11]. Like other ribozymes, the RNA components of the spliceosome are the major catalysts of splicing. It has been shown that human protein-free spliceosomes are capable of catalysing reactions that resemble both the first [12] and second [13] steps of trans-esterification reactions during splicing. The U-snRNAs are found throughout much of the eukaryotic kingdom and have the characteristic Sm-protein binding site, which is a conserved 8–10nt uridine-rich sequence flanked by two stem-loops. The structures of these snRNAs are also highly conserved in eukaryotes where they have been found.

To date many studies have shown that the U-snRNAs from a wide range of organisms share the same stem-loop folds [13]–[19]. The stem-loops within these snRNAs are important for interactions with snRNA-specific proteins. Each of the five snRNAs has a number of specific interacting proteins ranging from 4 in human to 10 in yeast [20]. However in deep-branching eukaryotes, the protein components are usually reduced [21]–[23]. Bioinformatic studies have shown that *Giardia* is likely to have most of the more conserved snRNA-associated major spliceosomal proteins although the less conserved ones may not have existed or may have been lost [6].

In addition to the highly conserved stem-loop structures of individual U-snRNAs, functional interactions between U-snRNAs, and between U-snRNAs and intron sites, are also conserved in eukaryotes. The 5′ sequence of the U1 snRNA base-pairs with the intron at the 5′- intron site, but is released before the actual catalysis proceeds. U4 snRNA is required for bringing the U6-snRNA (through base-pairing) into the catalytic centre, and is released before the first step of the splicing reaction [24]. U2, U6 and U5 snRNAs remain at the catalytic core throughout the splicing reaction. U2-snRNA loosely binds to the branch site of the intron in the active spliceosome, leaving the unbound branch-site adenosine, which can then interact with the phosphate group on the guanosine at 5′ of the intron through its 2′-OH group, and form an intron lariat. Three interactions between U2 and U6 were identified from studies of mammalian and yeast systems, and were shown to be required for splicing [25]–[28]. U5 appears to act as a scaffold RNA to hold the two exon-intron junction sites at appropriate orientation by its invariant loop [29]. We show here that these interactions between U-snRNAs, or with mRNAs, can be used to identify U-snRNAs.

The *Giardia* U5-snRNA was identified by computational analysis [8], and it folds into a conserved U5 secondary structure, although the primary sequence itself does not show homology with U5-snRNAs from other species. The U5-snRNP is required for both steps of splicing [30] and is the only snRNP found in all three types of splicing: major-, minor- and trans-splicing. The U5-snRNP-specific proteins Prp8 and Brr2 are also found in other deep-branching eukaryotes including *Trypanosoma brucei* [31] and *Trichomonas vaginalis* [32]. The Prp8 protein, a large, unique and highly conserved protein which has no obvious homology to other proteins, has a central role within the spliceosome and makes extensive protein-protein interactions throughout the various stages of pre-mRNA splicing [33].

Therefore, given the likely presence of U5, Prp8 and many other spliceosomal protein components as well as a few spliceosomal introns, it seems highly likely that *Giardia* has a functional major spliceosome containing all five spliceosomal snRNAs. The aim here is to test these predictions. We found that using information from some candidates helped identifying others; e.g. U2 helped find U6, which then helped identify a good U4 candidate. This leads to the conclusion that *Giardia* spliceosome may contain the basic components seen in more highly researched eukaryotes such as human, yeast, and plants.

## Results

### Prediction of a *Giardia* U1-snRNA candidate

Searching for U-snRNA candidates from *Giardia* based on primary sequence similarity failed, as expected, due to the observed low sequence similarity between *Giardia* and other well studied eukaryotes. However, the generally conserved structures of the U-snRNAs may allow a more advanced computational search for new U-snRNA candidates from the fully sequenced *Giardia* genome [34], [35]. Due to the reduced nature of the *Giardia* genome [21], [23], [35]–[37], it is not unlikely that some of the ncRNAs from *Giardia* also have been reduced in size and structure. For example, it has been shown that the U1 snRNA from *Trypanosoma brucei* is unusually reduced in that it only contains one stem-loop structure in contrast to the usual five stem-loops seen in other eukaryotes [38].

Besides structural information, sequence motifs of the U-snRNAs can also aid computational searches. It is known that U1-snRNA and U2-snRNA have direct interactions with introns through complementary nucleotide sequences; U1 binds to the 5′-intron splice site and U2 binds loosely at the branch site [39]. The three spliceosomal introns in *Giardia* [4], [5] share sequence similarities which indicate the presence of conserved 5′-, 3′- splice sites and the branch site [4]. Together with the conserved U-rich Sm-binding site, these sequence elements can be incorporated into a computational search for snRNAs from *Giardia*.

**Figure 1:**
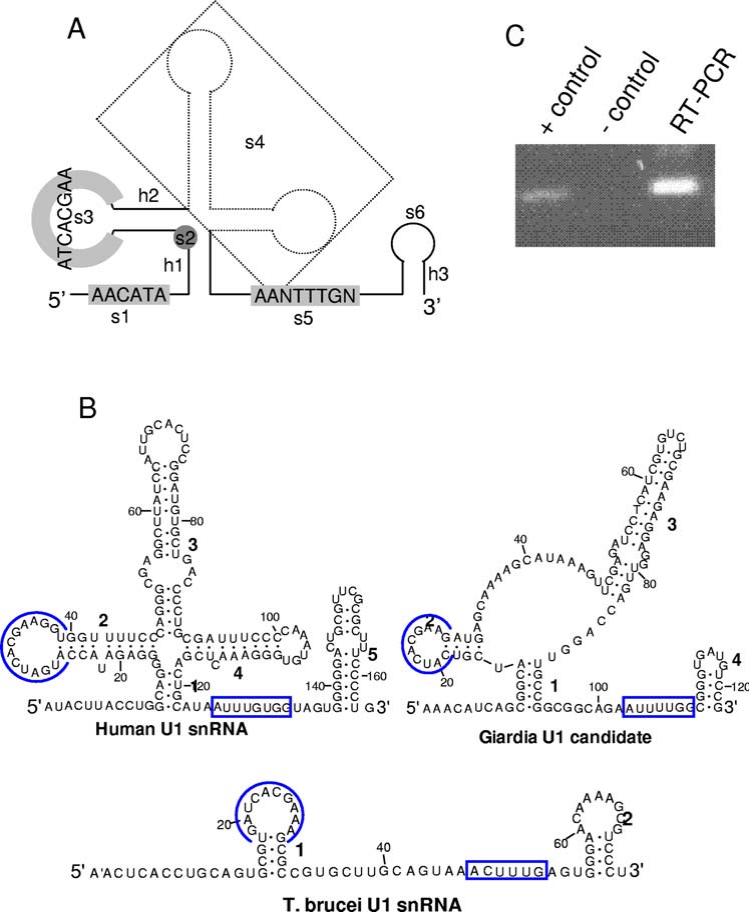
Identification of a *Giardia* U1-snRNA candidate. A. Proposed structure for writing the U1 descriptor file. The content in the U-1 descriptor cell can be visualized in this figure. “s” stands for strand and “h” stands for helix. The elements within the proposed U-1 structure are marked in order from the 5′-end to the 3′-end. The two stem-loops drawn as dotted lines are not compulsory in the proposed structure of *Giardia* U-1 candidate; therefore they are marked as a free-folding strand s4. B. The structures of Human, *T. brucei* and *Giardia*-candidate U1-snRNAs. The conserved loops among the human, *Giardia* and *Trypanosome* U1-snRNAs are indicated by the circles. The Sm-protein-binding sites are boxed. C. RT-PCR test showing high expression the of the *Giardia* U1-snRNA candidate. + control: PCR with genomic DNA. − control: PCR with total RNA without reverse transcription.

The computational prediction for U1-snRNA candidates was done using RNAbob (Materials and Methods). Since it was not known whether the U1-snRNA from *Giardia* was typical with conserved structure similar to human U1, or reduced like U1 from *T. brucei* [38], a relaxed model was set using the structural information from both the human and *T. brucei* U1-snRNA with human U1-snRNA as the upper limit of complexity and *T. brucei* U1-snRNA as the lower limit of complexity (Figure 1B). The stem-1 and stem-2 which were seen in both human and *T. brucei* are highly conserved at the loop sequence (Figure 1B). Therefore this loop sequence (conserved as “AUCACGAA”) is also incorporated into the search. Finally, a terminal stem which is present in both human and *T. brucei* was also used as a searching criterion. The descriptor file for U1 was written according to the proposed structure of the U1 candidate as shown in Figure 1A. This proposed structure is deduced based on known U1-snRNA structures together with information on the intron boundaries [4]. The descriptor file for searching U1 candidates is attached in supplementary information (Text S1).

This search produced only one output sequence, which has two copies in the *Giardia* genome, differing by only one base (see later). Their secondary structure has four stem-loop structures, two more stem-loops (stem-loop 3 in Figure 1B) than *T. brucei*. Thus the *Giardia* candidate is intermediate between the standard eukaryotic pattern as found in human, and the reduced one in *T. brucei*. Structural modelling based on the conserved structural and sequence elements as highlighted in the figure (Figure 1B) shows that it is a good U1-snRNA candidate.Expression of this *Giardia* U1-snRNA candidate was confirmed by RT-PCR (Figure 1C).

### Prediction of a *Giardia* U2-snRNA candidate

The same method was initially applied to search for U2 snRNAs from *Giardia*. However, this search did not give any results, probably due to the high degree of specificity required for constructing the descriptor file. Subsequently, a more general approach was tried. The new approach used the available sequences of U-snRNAs from Rfam [40] to search for the corresponding ncRNAs from the *Giardia* genome using the cmbuild and cmsearch programmes within the INFERNAL software package [41].

Two controls, one with U5 and the other with U1, were carried out to test the sensitivity of cmsearch. A control cmsearch using U5 snRNA was performed first. Using the model built from the alignment of 33 seed-sequences, the search resulted in 395 potential U5 sequences, including the previously reported U5 candidate [8]. This control strengthened the likelihood of obtaining a good candidate using cmsearch, but was clearly too general. A second control searching for U1 candidates was also performed. However, the putative U1 candidate found by RNAbob was not in the output which contains 29 sequences in total. This was not unexpected as the *Giardia* U1 candidate predicted by RNAbob has one stem-loop less than the conserved typical U1 structure (see Figure 1B), thus the search may have bypassed the *Giardia* sequence.

**Figure 2:**
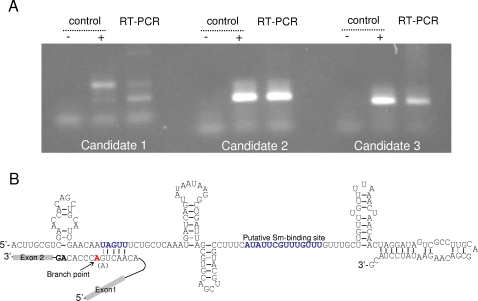
Identification of a *Giardia* U2-snRNA candidate. A. RT-PCR test for expression of the *Giardia* U2-snRNA candidates. The highly expressed candidate 2 was analysed further. − control: PCR with total RNA without reverse transcription. + control: PCR with genomic DNA. B. Structure of *Giardia* U2-snRNA candidate and its interaction with the branch-point intron region.

The cmsearch output for U2 produced only 5 hits. Blasting these hits against the *Giardia* genome database (http://www.giardiadb.org/giardiadb/) showed that 3 of the U2 hits lie within non-coding regions (including on the minus strand of protein-coding genes). Since the number of potential U2 candidates is small, RT-PCR analysis was carried out to test the expression of these hits, though the small number of hits may not cover all possible U2 candidates. Results (Figure 2A) clearly show that two of the three candidates (candidate-2 and candidate-3) are expressed and candidate-2 is highly expressed. Although candidate-3 is also shown to be expressed, it appears much less abundant than candidate-2. Structural modelling (Figure 2B) and sequence analysis show that candidate-2 is the most likely candidate for U2-snRNA.

In the active spliceosome, the bulged branch-site adenosine is crucial for the function of the spliceosome. It is expected that any potential U2-candidate from *Giardia* must have a sequence motif complementary to the branch site. The likely U2-candidate shown in Figure 2B contains a “UAGUU” motif which complements the 5′ of intron branch site “AACUG (or AACUA)”, but does not have upstream bases that can bind to 3′ of the branch-site adenosine (coloured red), thus instead of leaving the branch-site adenosine bulged this interaction leaves an open-end at the branch site. However this alteration of branch-site recognition may not induce any functional difference because the branch-site adenosine is still free to attack the 5′-guanosine phosphate. The overall sequence of this U2-snRNA candidate can fold into a typical U2-snRNA structure with the presence of a putative Sm-binding site, suggesting it to be a good candidate for U2-snRNA. This U2 candidate was used subsequently in searching for U6 and U4 snRNA candidates.

### Prediction of *Giardia* U6 and U4 snRNA candidates

Potential candidates for U6 and U4 snRNAs were first searched using INFERNAL. The outputs for U6 and U4 were large (1052 and 217 sequences respectively), and blasting the hits against the *Giardia* genome showed that 649 of the U6 hits and 114 of the U4 hits lie within non-coding regions. The large number of hits caused difficulty in further analysis; therefore an alternative method was used to search for U6 and U4 candidates based on the interactions between U2 and U6, and between U6 and U4.

**Figure 3:**
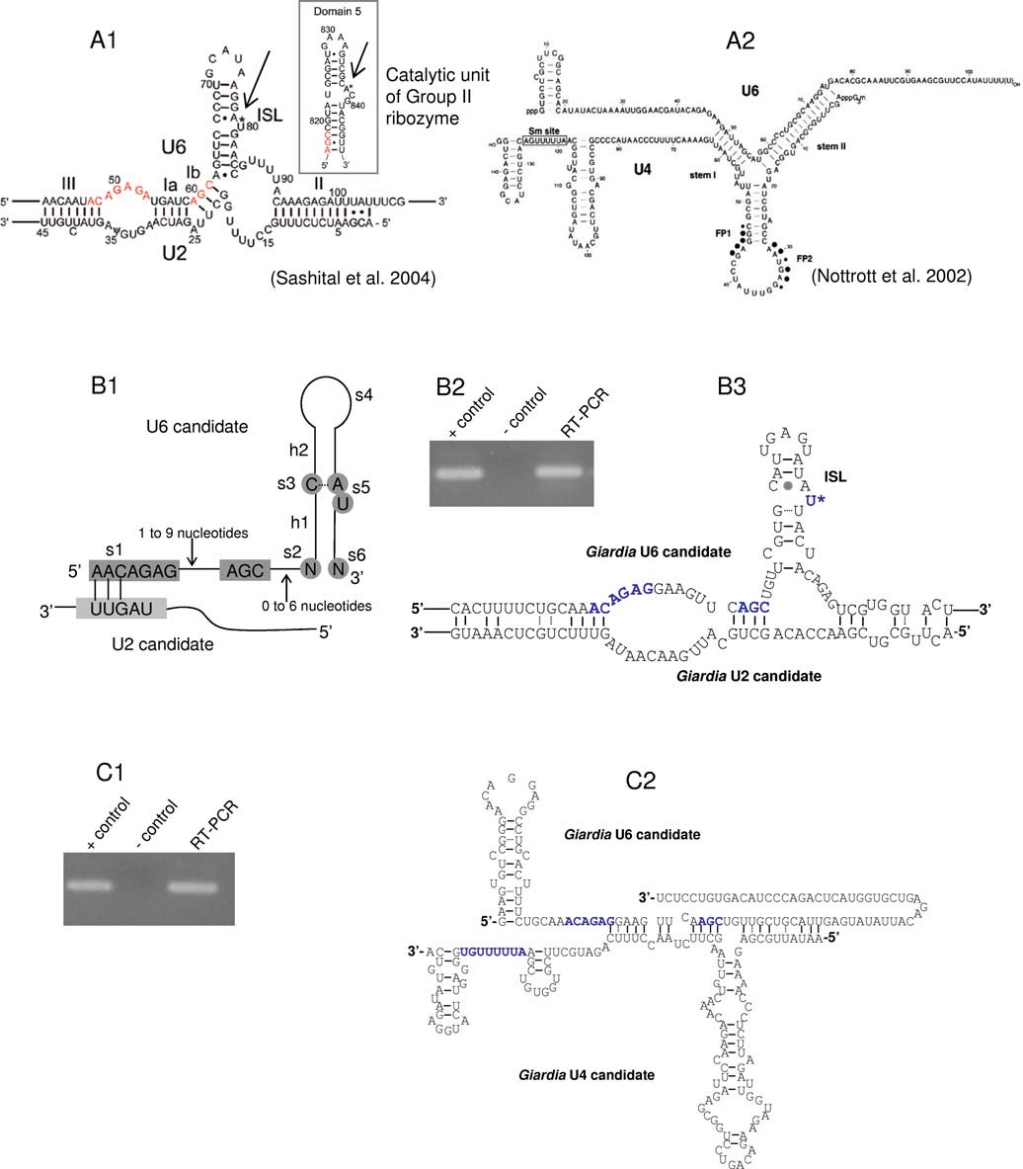
Identification of *Giardia* U6 and U4 snRNA candidates. A1. Structure of U2-U6-snRNA base pairing in *S. cerevisiae.* A2. Structure of U6-U4-snRNA base pairing in Human. B1. Visualization of the model for searching a U6-snRNA candidate. B2. RT-PCR test for expression of the U6 candidate. + control: PCR with genomic DNA. − control: PCR with total RNA without reverse transcription. B3. Interaction between *Giardia* U6 and U2 snRNA candidates. C1. RT-PCR test for expression of the U4 candidate. + control: PCR with genomic DNA. − control: PCR with total RNA without reverse transcription. C2. Interaction between *Giardia* U6 and U4 snRNA candidates.

It is known that conserved base pairings form between U2 and U6, and between U6 and U4 snRNAs during the dynamic process of splicing. These conserved base-pairings are shown in Figure 3A1-2. In the U2-U6 complex, the central region of U6-snRNA folds into an intramolecular-stem-loop (ISL) structure, which is highly conserved in the active spliceosome and juxtaposes the regions interacting with U2-snRNA [42]. The ISL has been shown to have important roles in the catalytic centre of the spliceosome with the uridine (indicated by * in the *S. cerevisiae* model shown in Figure 3A1) serving as a binding site for an Mg^2+^ ion during the catalytic step of splicing [43]. This uridine is seen in all but two U6-snRNAs from Rfam [44], and is usually situated below a “A·C” wobble base pair, which is readily protonated [43]. Mutation of the bulged uridine within U6-ISL has been shown to be lethal due to its resulted alteration of “A·C” wobble base pair which is important for melting the U6-ISL during structural rearrangement necessary for association with U4-snRNA [45]. It was later shown that base substitutions within the “A·C” wobble base pair disrupt Prp24 protein binding and reduce stability of the U4/U6 complex [46]. The structure of U6 ISL is highly similar to the catalytic stem-loop structure of Group-II ribozyme [13], [47] and it appears that this structure has been maintained through evolution of the splicing mechanism [48], [49].

In addition, two sequence motifs on the U6-snRNA are also conserved (coloured red in Figure 3A1). The “ACAGAG” is involved in base-pairing with the 5′-intron site and the branch site [47]. The invariant “AGC” tri-nucleotide is seen in all identified U6-snRNAs recorded in Rfam [44], and has both structural and functional roles during splicing [47]. A recent study also showed that the “ACAGAG” loop and “AGC” tri-nucleotide were binding sites of Mg^2+^ [50]. U6 and U4 also form extensive base-pairings [51] as shown in Figure 3A2. In this hybrid, the U6-snRNA has formed a 5′-stem-loop structure. Gathering all the sequence and structural features of U-snRNAs, Table 1 lists all the consensus properties used for searching U6 and U4 snRNA candidates.

**Table 1:** Criteria for searching U6 and U4 snRNA candidates in *Giardia*:

U-snRNA	Features
U6-snRNA	5′-stem-loop
	ISL with a bulged uridine, likely to be located below a “C-A” wobble pair
	ACAGAG motif
	AGC invariant tri-nucleotide
	Base-pairing with U2-snRNA on 5′ and 3′ of the ISL
U4-snRNA	GCT tri-nucleotide which base pairs with “AGC” tri-nucleotide of U6
	5′-sequence which base-pairs with U6 central region and sequence immediately after “GCT” which base-pairs with U6 near its 5′-stem-loop
	Sm-protein binding site (usually starts with ‘A’ followed by a number of ‘U’s and terminates with ‘G’)

A trial to search for a *Giardia* U6-snRNA candidate was carried out before U4 because there are more conserved features known for the U6-snRNA. A descriptor file (see supplementary information, Text S2) for the RNAbob programme was written based on the consensus features around the ISL, including the “AAC” motif which binds *Giardia* U2 at the 5′ end of the “ACAGAG” loop, and the “ACAGAG” motif and the “AGC” invariant tri-nucleotide which are two of the important characteristic features of U6-snRNA. The criteria used for writing the descriptor file can be visualized in Figure 3B1.

The descriptor file was then used to search against the whole genome sequence of *Giardia*. This gave 4 output sequences, of which two lie in non-coding regions. 40nt sequences upstream and downstream of the two output sequences were analysed. One of the two sequences has all the compulsory features of U6-snRNA (see Table 1), and was therefore identified as a candidate, even though this candidate is not found using INFERNAL-cmsearch. This is again possibly due to the low sequence conservation between *Giardia* U6 and those from most other organisms which were used as seeds for constructing the cmsearch model. Indeed low sequence conservation was the major problem in identifying *Giardia* ncRNAs and earlier trials to look for U6-candidates failed with sequence similarity search. RT-PCR testing has confirmed that this potential U6-snRNA candidate is highly expressed. Results are shown in Figure 3B2. Figure 3B3 shows the RNA complex formed by the U2 and U6 snRNA candidates from *Giardia*. Conserved sequence elements on the U6-snRNA candidate are coloured in blue.

Having used the U2 candidate to find U6, the U6 candidate was then used to search for a possible U4 candidate based on the conserved U6-U4 base-pairing feature shown in the human model in Figure 3A2. First, a potential U4-snRNA candidate was searched for from the 114 output sequences of INFERNAL-cmsearch. A few sequences from the cmsearch output contain a putative Sm-binding site but just one of them shows base-pairing with the U6-snRNA candidate. Expression of this sequence was tested by RT-PCR and the result (Figure 3C1) shows clear and high expression. The interaction between *Giardia* U6 and U4 snRNA candidates is shown in Figure 3C2. This structure (Figure 3C2) is consistent with the prediction that this is a good U4-candidate.

### Transcriptional patterns of the *Giardia* U-snRNA candidates

All five *Giardia* U-snRNA candidates are found in transcriptionally intensive regions (rich in open reading frames) of the genome; most of them overlap with protein-coding genes on the antisense strands. Gene overlapping is very common in the reduced genome of *Giardia*, and the lengths between protein-coding genes are generally short (less than 200bp) [35]. Previously identified non-coding RNAs in *Giardia* [52]–[55] are all located either in intergenic regions or overlap with protein-coding genes on the antisense strands. Therefore the locations of the U-snRNA candidates identified here are as expected. Except for the U1 candidate which has two copies with just a single base substitution between them, the other candidates all have a single copy in the genome. The locations of *Giardia* U-snRNA candidates in relation to the positions of nearby protein-coding genes are shown in supplementary information (Figure S1).

The upstream 100nt sequence for each U-snRNA candidate was extracted from the genome and analysed. It is known that in most eukaryotes, the U6-snRNA is transcribed by RNA Pol III [56], and the other four snRNAs are transcribed by RNA Pol II. The Pol II promoter sequence in *Giardia* has been shown to be roughly conserved [34], but there has been no Pol III consensus sequence for *Giardia* published to date. Our studies on the potential promoter elements in *Giardia* (unpublished data) shows that two “A”-rich motifs are likely to be the upstream promoter elements of Pol III. This information provides the basis for further analysis of the upstream sequences of the *Giardia* snRNA candidates.

The general eukaryotic U6 promoter contains an upstream “TATA-box” and also upstream enhancer elements [56], [57]. The upstream sequence of *Giardia* U6-snRNA candidate does not show a “TATA-box” motif. The upstream sequences of the other four U-snRNA candidates do not show strong signals of either Pol II or Pol III promoter elements. Absence of significant promoter signals indicates that these candidates may be examples of ncRNA genes being co-transcribed with adjacent protein-coding genes. The same feature is seen in more than half of the new ncRNAs candidates expressed in *Giardia* [54].

## Discussion

This study has found four good candidates for *Giardia* snRNAs through computational methods, and confirmed by RT-PCR analysis that they are highly expressed. A U5 candidate was reported earlier [8]. The sequences and genomic locations of five (U1, U2, U4, U5 and U6) *Giardia* U-snRNA candidates are listed as supplementary information (Text S3). Previously, only one (U5) snRNA had been identified in *Giardia*, so it had appeared possible that the ancestral spliceosome was mainly protein based, and that the catalytic role of snRNAs had evolved later in eukaryotic evolution. Now it seems likely that the last common ancestor of modern eukaryotes had a full spliceosome that functioned in much the same way as in plants, animals and fungi – that is, with functional snRNAs. Apart from the primary tests of expression, the *Giardia* U-snRNA candidates found here have not been extensively verified by biochemical methods. Two types of tests could carry this work on further. Detailed biochemical tests are now possible based on the candidates we identified however this is not yet straightforward. On the other hand, computational tests can now be done to search for snRNAs in related genomes, although they do not replace biochemical studies. *Trichomonas* and *Trypanosomes* would be good candidates because their genomes are complete. A very recent study has found U-snRNAs in *Trichomonas* [58], supporting our prediction that major spliceosomal snRNAs are likely to be common in all eukaryotes.

Combining sequence and structural information (which summarises conserved features of characterised ncRNAs) appears to be an efficient way of searching for unknown homologues of these ncRNAs in phylogenetically distant lineages. The structures of non-coding RNAs are important for their functions. Like proteins, non-coding RNAs with similar functions need not share extensive sequence similarities; however they generally fold into similar structures. A number of computational methods have been developed to fold a single RNA sequence [59], [60]; however, computationally predicted structures are often different from the true structures *in vivo*, because the folding of RNAs in the cell is usually associated with protein-cofactor binding and different metal ion associations. These conditions are hard to simulate. The structures of non-coding RNAs can be determined more reliably from other phylogenetically or functionally related non-coding RNAs which have been previously characterised.

The primary results from this study show that homologues of spliceosomal snRNAs are found in *Giardia*. Although evolutionary divergence between *Giardia* and other eukaryotes causes difficulties, combining different computational approaches based on available biological information has proved to be an efficient strategy. The snRNA candidates found in this study can be used as examples of snRNAs in evolutionarily deep-branching eukaryotes and help understanding of the evolution of the major spliceosome.

In this study, two software packages with different approaches were applied to search for the U-snRNA candidates in *Giardia*. The INFERNAL software uses covariance models [61] which optimize the alignment of an RNA sequence to a conserved RNA structure. INFERNAL is comparable to the HMMER package, which builds profile Hidden Markov models for searching for homologous protein sequences from a database. Eukaryotic U-snRNAs from Rfam have been annotated with the INFERNAL package with multiple alignments and conserved secondary structures. These alignments were used in searching for potential U-snRNAs from the *Giardia* genome. In contrast, RNAbob uses a user-specified input descriptor file which specifies the expected sequence and structural motifs, and searches for matching motifs in a sequence database.

Although we are not comparing these software packages, it was clear that the searching algorithms have differing sensitivities. The RNAbob programme used here is highly sensitive for searching RNAs with known structures and conserved sequence motifs, but requires enough information to construct a descriptor file. On the other hand, the INFERNAL software applies to more general searches using alignments of both sequences and structures of seed RNAs; however successful searches using this method largely depend on the prerequisite that the candidate RNA is highly conserved at both sequence and structural levels with the seed RNAs used for the search. In this study of *Giardia* U-snRNAs, it was not clear as to what degree *Giardia* U-snRNAs may be conserved with other known U-snRNAs, therefore it was highly desirable to employ two search methods using different approaches to find candidates efficiently.

It is important to rely firstly on the biological information of the particular candidate before choosing a computational method. Using different computer programmes can increase the likelihood of finding the expected RNA candidate, although the outputs of different search methods do not always overlap. In all, our identification of *Giardia* snRNA candidates demonstrates an efficient way of searching for novel non-coding RNAs by combining biological information with computational methods. This approach is especially applicable where large scale biochemical isolation is not feasible. Results from this study also indicate that major spliceosomal snRNAs are highly likely to be present in ancestral eukaryotes, because they are found in all eukaryotes including the deep-branching lineages such as *Giardia*. This finding, if confirmed by future work, supports the highly distinctive nature of the eukaryotic cell [1].

## Materials and Methods

### Computational methods

The RNAbob source code was downloaded from http://genome.wustl.edu/eddy/#rnabob/, and modified to run under Windows. This programme uses a descriptor file which specifies the structure and sequence motifs of the RNA to be searched, and looks for matching candidates from a sequence database. The descriptor file for U1-snRNA was constructed using the information available for *Giardia.* The search model was set so that the expected output would have the 5′-intron site recognition sequence “AACAUA”, which complements the “UUGUAU” sequence at the 5′ end of the intron. The Sm-binding sequence was set to “AANUUUGN” where N indicates an uncertain nucleotide. All the “U”s are written as “T”s in the descriptor file for searching in a DNA genome.

In the descriptor file, lines starting with “#” are comments. The “strands” and “helices” elements within the proposed structure are listed in order, and each of them is then specified. “N” represents an uncertain nucleotide which is definitely present and “*” represents an optional nucleotide. [ ] indicates the maximum number of nucleotides present. Optional stems were replaced by long strands. The numbers immediately following an element (s1, h1 etc.) described indicate number of mismatches allowed. For example “h1 0:0” shows that no mismatches are allowed in the helix h1.

The INFERNAL software was downloaded from http://infernal.janelia.org/, and alignments of snRNAs from various species were downloaded in Stockholm format from the Rfam database [44]. The INFERNAL programmes were run under the Linux operating system with the default settings.

The alignments of U1, U2, U4, U5, and U6 were downloaded from Rfam [44] and covariance models for these alignments were built using the INFERNAL-cmbuild programme. Searching for potential U-snRNAs from the *Giardia* genome was done by the INFERNAL-cmsearch programme. An output hit from cmsearch consists of an alignment and a score. By default, scores above 0 are considered as hits.

### *Giardia* total RNA preparation

Total RNA was extracted from *Giardia* WB strain Trophozoites grown in TY1-S-33 media. Cells were collected by centrifugation (10 min, 3000rpm, 4°C). RNA extraction was performed using Trizol reagent (Invitrogen, Cat# 15596-026) according to the manufacturer's instruction. The extracted RNA was dissolved in sterile double-distilled water. The purified RNA was treated with DNase-I (Roche Cat# 04 716 728 001) for 1 hour and purified by phenol/chloroform extraction and ethanol precipitation.

### RT-PCR

All the RT-PCR reactions were performed using the Thermoscript cDNA synthesis kit (Invitrogen, Cat# 11146024). Total RNA treated with DNase was mixed with the corresponding reverse primer and dNTPs. The mixture was heated to 85°C for 2 min and cooled gradually. Then a mixture of reaction buffer, RNaseOUT and reverse transcription enzyme was added. All RT reactions were carried out for 1 h at 55°C and then heated to 85°C to inactivate the enzyme. 2 µl RT reaction was taken out to serve as the template for the downstream PCR reaction. Results were analyzed on 2% agarose gels. Primers used for testing expression of the U2, U4 and U6 snRNA candidates are listed below:

**Table:** 

GU1_cand_1_F	AAACATCAGCGGCATCGTCA
GU1_cand_1_R	CGGACATCACCCGCCAAAA
U2_cand_1_F	CTATATGATGACTATTAATAGTAAGTTTAAAGA
U2_cand_1_R	GTTGCTTCTAATATATAGTGAGGGA
U2_cand_2_F	ACAGCTGCATTGAACAATAGTTTCT
U2_cand_2_R	CAAGGCGACTATCCTAGTTG
U2_cand_3_F	TCA CCT CAC ATG ATT TGG TGA
U2_cand_3_R	TACATTTCTGCGGGGAGTCT
Likely_U6_F	AGTGTCCGGGAACAAGTGAG
Likely_U6_R	TAGGGTCTGAGTACCACGAC
Likely_U4_F	TATTGCGAGAAAACCCTCTTAG
Likely_U4_R	CCCACAAAAATTCGACACCAC

## Supporting Information

Text S1 U1 descriptor file
(0.00 MB TXT)

Text S2 U6 central region descriptor file
(0.00 MB TXT)

Text S3 Sequences and genomic locations of Giardia snRNA candidates
(0.00 MB TXT)

Figure S1 Locations of Giardia snRNA candidates In this figure, black arrows indicate the direction of protein-coding-gene transcription and grey arrows indicate the direction of Giardia U-snRNA candidates. The lengths of arrows are not proportional to the actual lengths of transcripts, because the mRNA transcripts are much longer than the snRNA candidates.
(0.78 MB TIF)
